# Replacing the Draize eye test: Impedance spectroscopy as a 3R method to discriminate between all GHS categories for eye irritation

**DOI:** 10.1038/s41598-018-33118-2

**Published:** 2018-10-09

**Authors:** C. Lotz, L. Kiesewetter, F. F. Schmid, J. Hansmann, H. Walles, F. Groeber-Becker

**Affiliations:** 1 Department Tissue Engineering & Regenerative Medicine (TERM), University Hospital Würzburg, Würzburg, 97070 Germany; 2 Translational Center for Regenerative Therapies, Fraunhofer Institute for Silicate Research, Würzburg, 97070 Germany

## Abstract

Highly invasive animal based test procedures for risk assessment such as the Draize eye test are under increasing criticism due to poor transferability for the human organism and animal-welfare concerns. However, besides all efforts, the Draize eye test is still not completely replaced by alternative animal-free methods. To develop an *in vitro* test to identify all categories of eye irritation, we combined organotypic cornea models based on primary human cells with an electrical readout system that measures the impedance of the test models. First, we showed that employing a primary human cornea epithelial cell based model is advantageous in native marker expression to the primary human epidermal keratinocytes derived models. Secondly, by employing a non-destructive measuring system based on impedance spectroscopy, we could increase the sensitivity of the test system. Thereby, all globally harmonized systems categories of eye irritation could be identified by repeated measurements over a period of 7 days. Based on a novel prediction model we achieved an accuracy of 78% with a reproducibility of 88.9% to determine all three categories of eye irritation in one single test. This could pave the way according to the 3R principle to replace the Draize eye test.

## Introduction

The human eyesight is one of our most relied senses to perceive our environment. To ensure public health, all chemicals need to be evaluated for their potential to cause eye irritation. For this reason, an *in vivo* test system was developed by John Draize in 1944 to assess eye irritation^1^. In this Draize eye test, a test substance is applied in one eye of an albino rabbit and the effects, e.g. chemosis, conreal opacity, irititis, and conjunctival redness, are analyzed over a period of 21 days. Based on a scoring system, the tested chemical is then classified according to the United Nations globally harmonized systems (UN GHS) categories. Substances are labeled as (I) causing no irritation: no category, (II) serious eye damage: category 1 or (III) eye irritation: category 2. Hereby, category 2 can be further subcategorized as category 2A if an effect is reversible within 21 days or category 2B in case the caused effect persists not more than 7 days. Although the original test was improved following the 3Rs principle by the use of topical anesthetics and analgesics to reduce animal suffering, there are still ethical concerns and scientific limitations especially in regard to over-prediction and inter-laboratory variations^2,3^. These pitfalls and ethical issues drove a change in legislation to develop alternative test methods that do not include the use of test animals^4^.

For the endpoint of eye irritation, several *ex vivo* and *in vitro* test methods have been developed and incorporated into Organisation for Economic Co-operation and Development (OECD) guidelines^5–9^. The developed tests comprise a wide variety of models and analysis tools to identify category 1 and no category substances. Yet, no single test method can completely replace the Draize eye test and particularly category 2 substances causing reversible effects cannot be detected by the available test methods OECD TG 437,438,460,491 and 492. More recently, an *in vitro* procedure employing a reconstructed human cornea-like epithelium (RhCE) based on epidermal keratinocytes was accepted as OECD test guideline 492^9^. The assay evaluates cytotoxicity of a test substance in a RhCE, measured by the 3-(4,5-dimethylthiazol-2-yl)-2,5-diphenyltetrazolium bromide (MTT)-assay^9^. Nevertheless, the test procedure is limited to the discrimination between no category versus category 1 and category 2 substances^9^. Thus, no single alternative test method is capable of identifying all UN GHS categories since the methods cannot distinguish between category 1 and category 2 substances.

The currently available models are either from non-human species or based on a different, non-corneal cell origin such as skin keratinocytes for the RhCE. The anatomical and molecular differences between species lead to false positive and false negative results in pharmaceutical and toxicological tests like the Draize eye test^3^. By using human-derived tissue models this critical pitfall can be avoided. However, no systematic study has been performed if and to what extend skin-derived cells influence the outcome of toxicological tests in comparison to cornea-derived cells. Therefore, a cornea epithelial model based on human corneal cells should be employed that mimics the histological morphology and the molecular network of native human cornea in comparison to the state of the art skin derived models.

Another drawback of the existing *in vitro* test system is the analysis via the destructive MTT-assay. As an endpoint-test, the enzymatic conversion of the yellow dye MTT into a blue MTT formazan salt by cells with an active metabolism render the model unusable for further testing^10^. Thus, no reversible effects can be detected. In addition, category 2 substances comprise subtle changes, which do not necessarily lead to cell death but might only decrease tissue integrity by the loss of cell-cell or cell-matrix connections^11^. Hence, the measurement of the epithelial barrier might be an additional parameter to refine current testing strategies. To investigate cellular barrier-functions the measurement of the transepithelial electrical resistance (TEER) was developed. However, the employed technical systems were mostly used for two-dimensional cell layers and are only of limited value in the assessment of multicellular barriers^12^. By using impedance spectroscopy capturing the complex alternating current resistance over a broad frequency spectrum, e.g. from 1 Hz to 100 kHz, one can identify small changes in the barrier also in three-dimensional tissue models caused at certain frequencies and enable more sensitive measurements to detect changes through mildly irritant substances.

This study aims to adapt an eye irritation test by adjusting the model and the analysis tool to identify all GHS categories. At first, a new model derived from human corneal epithelial cells was developed and compared to an in-house developed cornea-like model based on skin keratinocytes and a standard reconstructed human epidermis (RHE) regarding epithelial marker localization and sensitivity to hazardous chemicals. To further refine the assessment of eye irritation, impedance spectroscopy was employed to monitor key changes non-destructively in the tissue integrity over 11 days. We hypothesize that, by the combination of an *in vitro* model with non-destructive impedance spectroscopy, a novel test procedure can be established to replace the animal based Draize eye test.

## Results

### Cornea-specific marker expression in the RCE model

**Figure 1:**
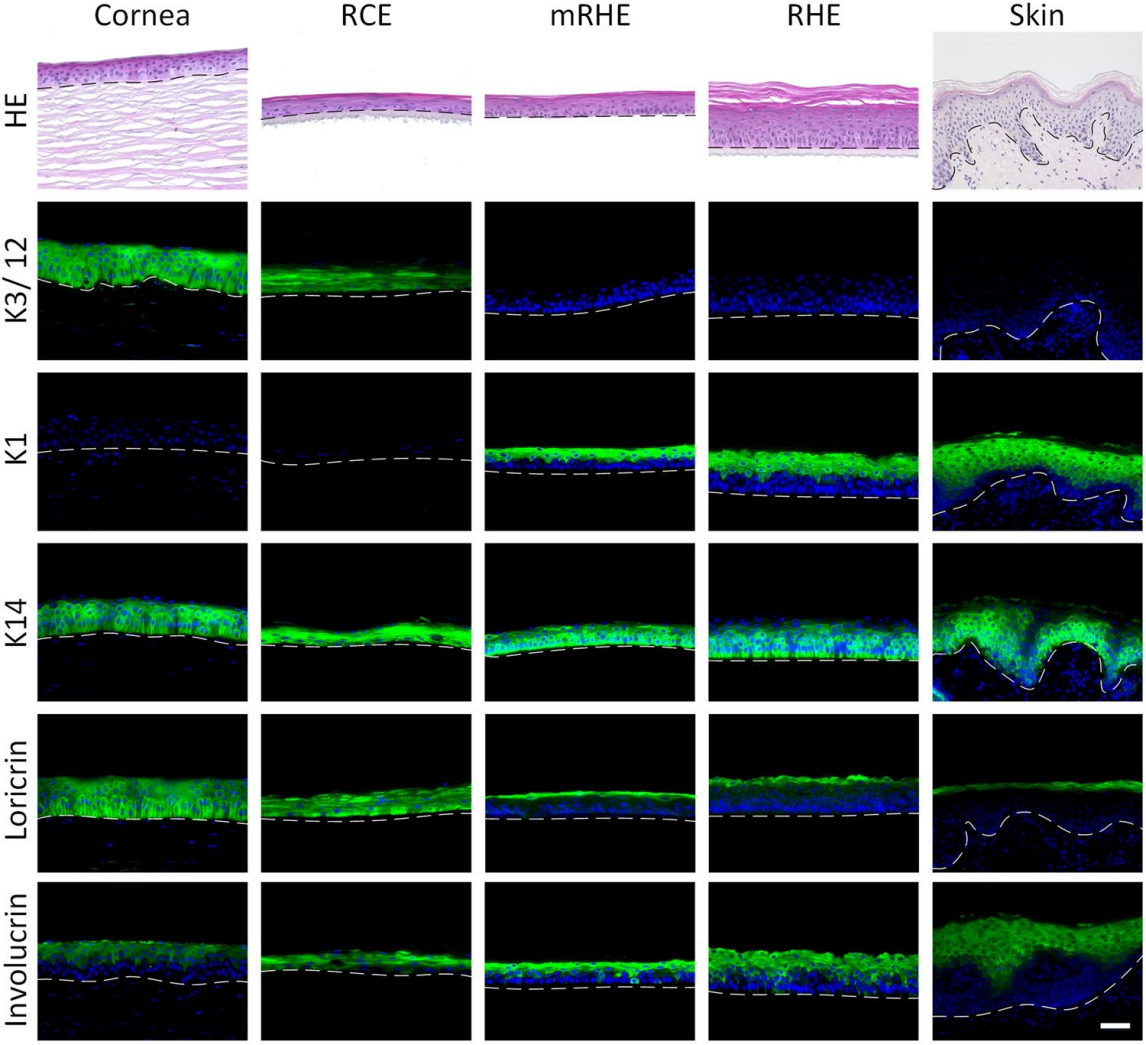
Primary cell origin defines marker localization in tissue models. Histological characterization of cornea models. Hematoxylin and eosin (HE) staining of native human cornea (Cornea), reconstructed human corneal epithelium (RCE), modified reconstructed human epidermis (mRHE), reconstructed human epidermis (RHE) and native human skin (Skin). Tissue-specific proteins cytokeratin 3/12 (K3/12), cytokeratin 1 (K1), cytokeratin 14 (K14), loricrin (Loricrin) and involucrin (Involucrin) were visualized by immunofluorescence staining. Cell nuclei were labeled with DAPI. Dotted lines show the basal membrane and the scale bar indicates 20 µm.

Human primary cells are an important source to generate organotypic tissue models for preclinical research. They comprise the *in vivo*-like protein expression and metabolism to rebuild the native tissue’s anatomy and function^13^. To date, human epithelial cells from skin are employed to create tissue models for eye irritation according to the OECD test guideline 492. However, epidermal cells from skin differ from the corneal epithelial cells of the eye in protein expression and metabolism^14^. To estimate whether a corneal cell source would be suitable for the assessment of chemicals for eye irritation, primary human epithelial cells from cornea and epithelial cells from skin were used to generate corneal tissue equivalents. The native cornea epithelium, in comparison to the epidermis of the skin, is a non-keratinized stratified squamous epithelium. For comparison of the models’ anatomy, histological analysis of the reconstructed human cornea epithelium (RCE), modified reconstructed human epidermis (mRHE), reconstructed human epidermis (RHE), native skin and native cornea was performed. The RCE and the mRHE show the same histological features as the native human cornea with 4 to 6 cell layers and little or no cornified layer. In contrast, the RHE features more cell layers and a keratinized layer comparable to the epidermis *in vivo* (Fig. 1). To further elucidate the molecular framework of the test models, an immunohistochemical staining of tissue-specific markers was performed. The ubiquitous epithelial marker cytokeratin 14 (K14) was expressed in the native tissues as well as in all of the three generated models. However, the cornea-specific markers cytokeratin 3 and 12 (K3/12) were only found in the native cornea and in the RCE. Cytokeratin 1 (K1), an epidermis marker, was visualized in the native skin, the RHE and the mRHE model but not in the RCE model. Moreover, the markers involucrin and loricin were analyzed to assess if a cornified layer was formed. The native cornea and the RCE showed a homogenous distribution of loricrin throughout all cell layers. On the contrary, locrin was limited to the stratum granulosum and the stratum corneum in the native epidermis, the RHE and the mRHE. Involucrin, as a substrate protein of transglutaminases, could be identified in the suprabasal layers of all studied epithelia (Fig. 1).

### Highly sensitive impedance spectroscopy can be employed to measure tissue barrier function

**Figure 2:**
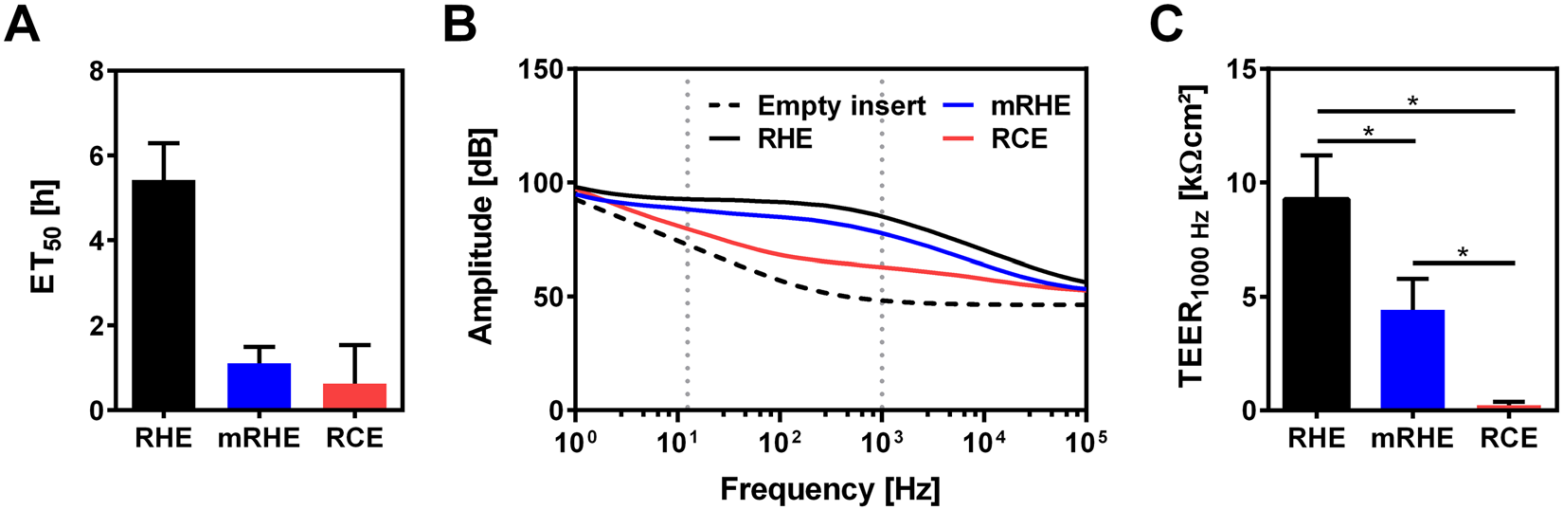
*In vitro* cornea models display a lower barrier function compared to reconstructed human epidermis. (**A**) ET_50_ assay to evaluate barrier properties of reconstructed human epidermis (RHE), reconstructed human corneal epithelium (RCE), and modified reconstructed human epidermis (mRHE) (n = 3; mean value ± SD). (**B**) Amplitude of the impedance of RHE, RCE, and mRHE in comparison to an empty insert. (**C**) Transepithelial electrical resistance measured at 1000 Hz and multiplied by the culture area of RHE, RCE, and mRHE (n = 30; mean value ± SD; p < 0.05).

To assess if the specific cell origins and marker expressions translate into different barrier functions, an ET_50_ assay was employed. Through the challenging of the models with the irritative substance Triton X-100 for defined periods, the time at which half of the cells in the model are viable can be measured as an indicator for the barrier function of the models. RHE with an ET_50_ value of 5.4 ± 0.9 hours is much less sensitive to Triton 100-X than the 1.1 ± 0.4 hours of the mRHE and 0.6 ± 0.9 hours of the RCE. In the ET_50_ assay, no significant discrimination could be found between the RCE and mRHE (Fig. 2A). As an additional method to determine the tissue barrier, impedance spectroscopy was performed. By measuring the impedance over a frequency range from 1 Hz to 100 kHz, specific courses of the amplitude of the impedance and the phase angle can be analyzed in the so called Bode plots. Curve shapes altered both with type of tissue and culture time and each tissue forms a distinct pattern mainly between 1 Hz and 1 kHz compared to the course of an empty insert membrane (dotted line in Fig. 2B). For the RHE, the amplitude showed a more stable plateau between 10 Hz and 1 kHz, whereas the amplitude declines faster for the mRHE and RCE models. Since differences between the models were only poorly visible at 12.5 Hz, the frequency at which the Millicell-ERS2 hand electrode determines the TEER value, we chose 1000 Hz as the reference frequency for our study. In doing so, a new TEER value was defined by extracting the impedance amplitude in Ω at 1000 Hz and by multiplying the culture area of the model (0.6 cm^2^), returning the TEER_1000 Hz_ value in Ωcm^2^ used for analysis of all impedance data throughout the study.

Measuring the TEER_1000 Hz_ resulted in significant differences between the RHE and the mRHE but also between the mRHE and the RCE (Fig. 2C), which could not be detected by the ET_50_ assay (Fig. 2A). The RHE thereby had the highest TEER_1000 Hz_ of 9268 ± 1929 Ωcm^2^ followed by the mRHE with 4422 ± 1366 Ωcm^2^ and the RCE with the lowest TEER_1000 Hz_ of 232 ± 142 Ωcm^2^, showing a similar trend as in the results of the ET_50_ assay. (Figure 2C).

### RCE demonstrates best eye irritation prediction in MTT-assay

**Figure 3:**
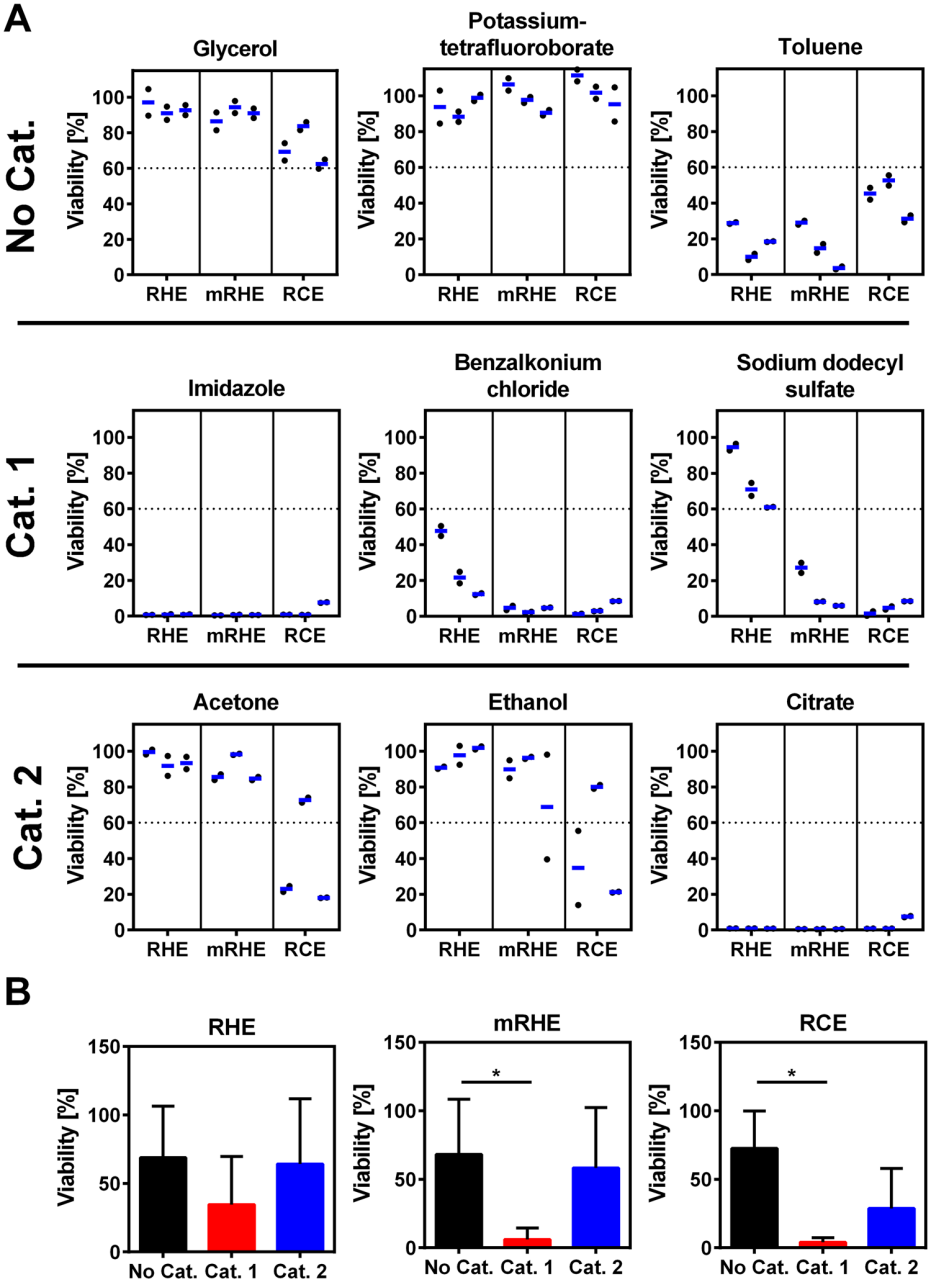
MTT-based eye irritation test cannot distinguish between all eye irritation categories. Eye irritation test with reconstructed human epidermis (RHE), reconstructed human corneal epithelium (RCE), and modified reconstructed human epidermis (mRHE) according to OECD TG 492. (**A**) The viability was normalized to the negative control. Three different test substances of each GHS category for eye irritation were applied (three test runs with duplicates). (**B**) To assess the ability of the different models to distinguish between the eye irritation categories, test substances were grouped to the respective GHS category for eye irritation. Shown are mean values and standard deviation of the substances from each category. The viability was normalized to the negative control (n = 9; mean value ± SD; p < 0.05).

In the next step, the models and the TEER measurements were implemented in an eye irritation testing protocol based on the OECD TG 492. Therefore, the models were measured before and after the application of the nine test chemicals, three of each GHS category (Table 1 and Fig. 3). To challenge the new protocol, toluene and citrate were selected on the basis that they cannot be predicted indisputably with the state-of-the-art tests. Phosphate-buffered saline was employed as negative control and 5% sodium dodecyl sulfate as positive control. As defined in the published test protocol, 60% viability was used as a cut-off value to distinguish irritative and non-irritative substances^9^. Technical duplicates were employed, similar as in the validation study of the accepted test method implemented in the TG 492. On top, three independent test runs with tissues generated from cells of different donors were performed to ensure robustness.

At first, the performance of the models was tested using the standard MTT-analysis. RHE had a higher viability compared to the mRHE and the RCE, when category 1 and category 2 substances such as sodium dodecyl sulfate and acetone were applied. The mRHE led to reproducible test results in the three test runs and the technical duplicates with one exception in case of ethanol. In comparison to the RHE, the mRHE demonstrated a stronger decline of viable tissue for category 1 substances. The RCE was the only model that showed significant reduction of viability for category 2 substances (Fig. 3A,B). While the RCE had the same outcome for the technical replicates, one donor-specific outlier was found in the test results for acetone and ethanol, though. The unharmonized test substances toluene and citrate reduced the viability of all models below the threshold of 60% (Fig. 3). Taken together, all three models achieved the same specificity of 66.7% in identification of irritants. However, the RHE showed the lowest sensitivity of 50% and lowest accuracy of 55.7%. The mRHE prediction was better with a sensitivity and an accuracy of 66.7%. The best results were achieved with the RCE with 100% sensitivity and 88% accuracy employing the MTT-based test method derived from the OECD TG 492 protocol.

To evaluate the capacity of the models to distinguish between the different categories, statistical analysis was performed taking the combined results from all test substances in each category (Fig. 3B). No significant difference between the groups could be found for the RHE. Meanwhile, the mRHE and the RCE were able to distinguish between no category and category 1. Additionally, the RCE showed the biggest differences between irritants and non-irritants in the analysis.

### TEER measurements improve sensitivity in RHE and mRHE

**Figure 4:**
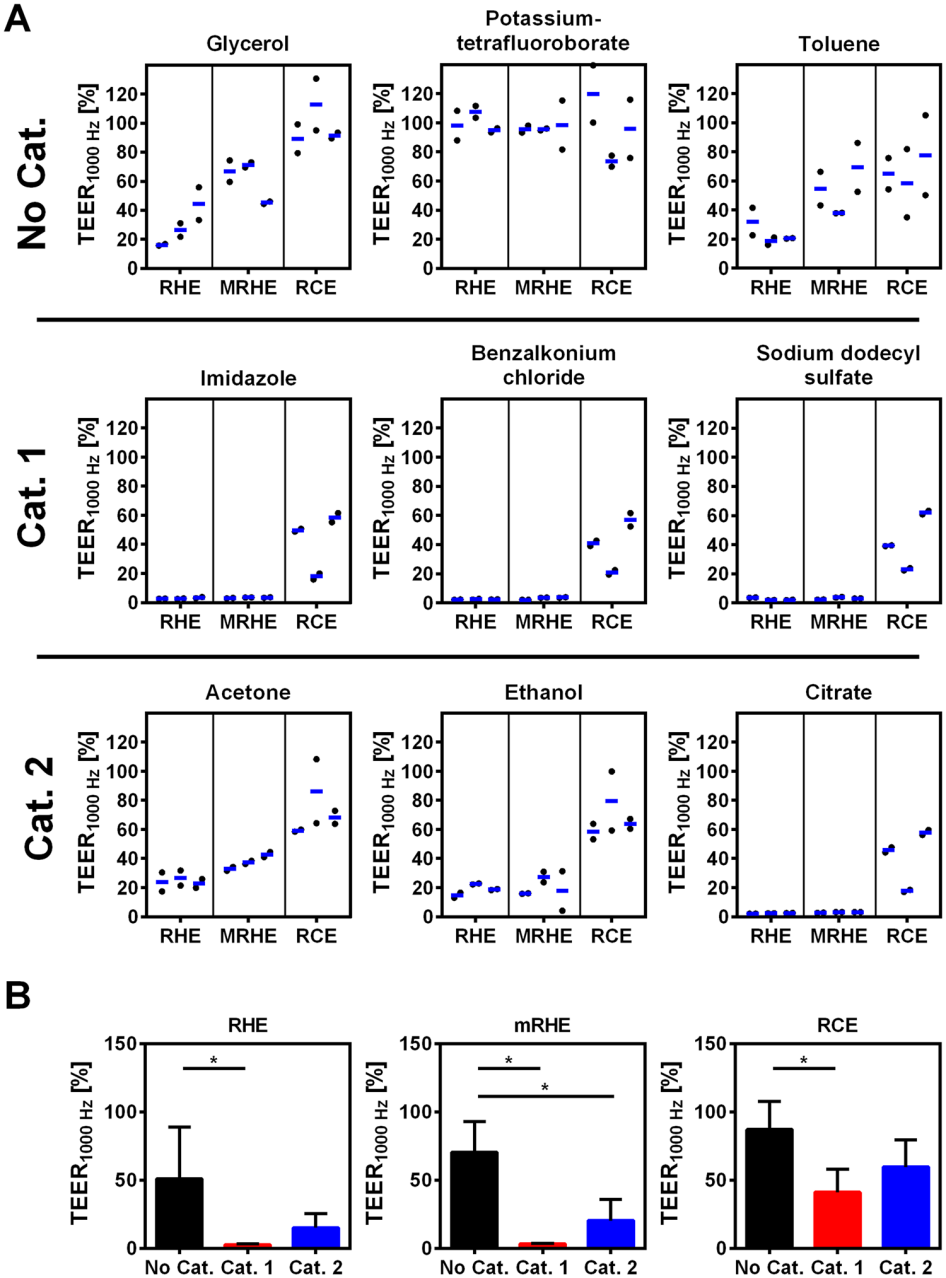
TEER_1000 Hz_ improves sensitivity of eye irritation testing. The impedance of reconstructed human epidermis (RHE), reconstructed human corneal epithelium (RCE), and modified reconstructed human epidermis (mRHE) was measured. (**A**) The TEER_1000 Hz_ was normalized to the negative control. Three different test substances of each GHS category for eye irritation were applied (three test runs with duplicates). (**B**) To assess the ability of the different models to distinguish between the eye irritation categories, test substances were grouped to the respective GHS category for eye irritation. Shown are mean values and standard deviation of the substances from each category. The TEER_1000 Hz_ was normalized to the negative control (n = 9; mean value ± SD; p < 0.05).

TEER measurements enable the detection of differences that could not be observed via the MTT-assay (Fig. 2C). To allow comparability between the different models, the TEER_1000 Hz_ was normalized to the negative control. Glycerol showed a decrease in the tissue integrity, whereas toluene induced only a moderate drop in the MTT data. Furthermore, the impedances of the category 1 chemicals imdiazole, benzalkonium chloride and sodium dodecyl sulfate decreased below 20% of the intact tissue in the RHE and mRHE. The values of category 2 chemicals acetone and ethanol fell between 20% and 50%. Citrate resulted in similar shifts as the chemicals of category 1. The RCE values were overall high and did only show minor changes between the different chemicals of the categories (Fig. 4A).

To address the question whether the TEER_1000 Hz_ can discriminate between the respective GHS categories, the results from each category were combined. By using the TEER measurements, the predictive capability increased for RHE and mRHE. The RHE was able to identify differences between no category and category 1 and the mRHE between no category and category 1 as well as among no category and category 2. However, the predictive capability did not increase for the RCE-based test protocol (Fig. 4B). Although there are clear differences between irriating and no non-irritating substances, it should be noted that a clear differentiation between category 1 and 2 is not possible. Thus, in the second step we added repeated TEER measurements over 11 days to gain also information concerning the persistence of irritating effect.

### Repeated TEER measurements allow for distinction between the GHS categories

**Figure 5:**
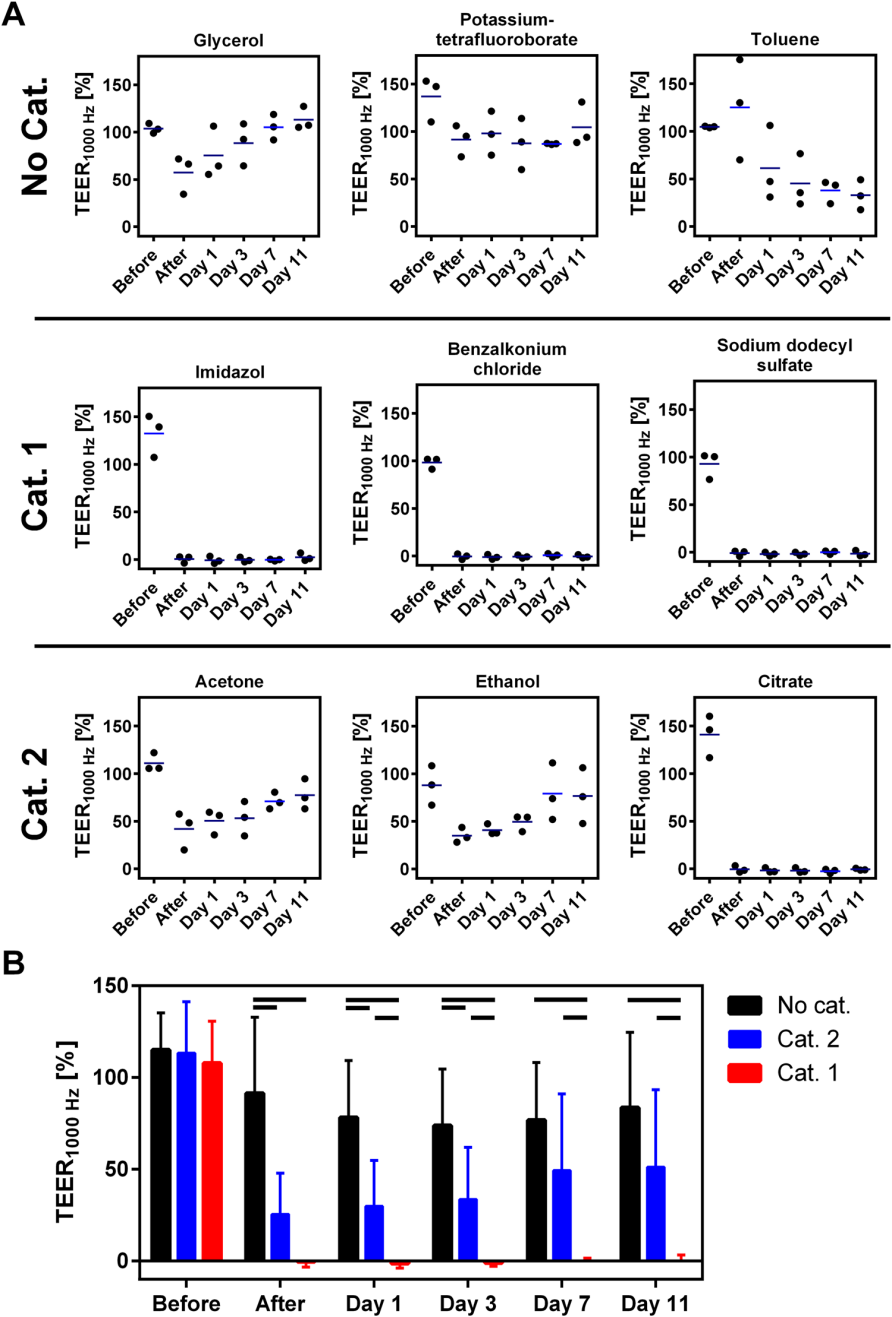
Repeated TEER_1000 Hz_ measurements allows to distinguish between all eye irritation categories. Ability to distinguish eye irritation categories by repeated TEER_1000 Hz_ measurements. (**A**) Eye irritation test with repeated TEER_1000 Hz_ measurements normalized to the control of the modified reconstructed human epidermis (mRHE) during a time period of 11 days after application of the chemicals (n = 3;. mean value). (**B**) To assess the ability of the different models to distinguish between the eye irritation categories, test substances were grouped to the respective GHS category for eye irritation. Shown are mean values and standard deviation of the substances from each category. (n = 9; mean value ± SD; p < 0.05). (**B**) (n = 9; p < 0.05).

To assess whether an effect is reversible, we measured the TEER_1000 Hz_ before the application of the test substance, directly after the application and on day 1, 3, 7 and 11. Due to the high predictive capability in the TEER_1000Hz_-based test protocol and less donor deviation compared to the RCE, the mRHE was chosen as the best suited model to perform repeated TEER measurements. Of the no-category substances, glycerol showed an initial decrease of TEER_1000 Hz_ value between 29% and 66%. Subsequently, the impedance recovered over time and reached over 100% at day 11. The TEER_1000 Hz_ of potassium tetrafluorobate stayed at around 100% compared to the negative control over the whole time period. For toluene as a non-harmonized substance, values decreased only slightly directly after the application. However, the effect was persistent and led to a continuous decrease over the 11 days resulting in TEER_1000 Hz_ values below 50%. Category 1 substances, in contrast, decreased the TEER_1000 Hz_-value below 6% of the initial level showing no recovery during the 11 days. The TEER_1000 Hz_ of mildly irritating category 2 substances with the exception of citrate dropped below 50% after the application and recovered to levels slightly above 50% during 11 days (Fig. 5A). Interestingly, the impedance of citrate showed a comparable course to category 1 substances and decreased to less than 8% with no signs of recovery.

To verify that the repeated measurements allow for distinguishing between the different categories, statistical analysis was performed at each measurement point (Fig. 5B). Before the application of the test substances, no significant differences can be found. However, after application of the chemicals the pairs no category versus category 1 and no category versus category 2 can be differentiated as shown before. Following a post-incubation period of 1 and 3 days, all eye irritation categories show significant different TEER_1000 Hz_ values. After day 7, models challenged with no category and category 2 test substances reached similar tissue integrity and did not differ significantly, while still being significantly different to category 1 (Fig. 5B).

## Discussion

Tissue engineering was developed as a technology to replace organ defects. However, since functional tissue equivalents are created, the same technology is applicable for the creation of organotypic tissue models for toxicity testing or pre-clinical research. Using these models, novel standardized test methods were developed that in some cases reached sufficient predictivity to become part of internationally valid OECD guidelines^6,8,9,15^. Despite these advances, some toxicological endpoints still require additional animal-based test methods for a meaningful prediction. Although the recent advances in the development of tissue engineered corneal models led to the new OECD TG 492 that can discriminate between irritants and non-irritants, until today no stand-alone test procedure has been developed to fully replace the Draize eye test. In this systematic study we could show that the cornea derived models, while having a native molecular pattern, demonstrate no significant improvement over the skin based models for the prediction of eye irritation. However, through the use of the TEER_1000Hz_ in combination with the mRHE we could distinguish between all categories of eye irritation by repeated measurements over 7 days of the treated models.

To replace the Draize eye test we hypothesized that a corneal model in combination with a non-destructive measurement should allow to distinguish all GHS categories of eye irritation *in vitro*. Hence, in this study we generated cornea epithelium models based on either skin derived keratinocytes as a reference to the state of the art models or primary cornea epithelial cells and compared these models in regard of morphology and the localization of cornea and skin-specific cellular markers. One of the essential differences between cornea and the skin is the cornification of the epithelium. The cornified layers of the epidermis function as a tight barrier formed by dead tissue to protect us from physical, chemical, and biological hazards. The cornea on the other hand needs to be transparent to allow light to reach sensory cells in the retina and thus does not comprise keratinized layers. Without this strong physical barrier, the cornea is more vulnerable. So to mimic the situation, a model system should not feature a cornified layer in order to decrease the sensitivity^14^.

The cornea epithelial cells show no cornification in the RCE model in comparison to the epidermal keratinocytes from the RHE. Yet, keratinocytes from the epidermis and cornea epithelium are closely related by the origin from the ectoderm and it could be shown that cornea keratinocytes transdifferentiate to epidermal keratinocytes and vice versa^14,16^. Therefore, both cell origins seem suitable for generating models for eye irritation testing. This close relationship between both cell types was recently used to generate the EpiOcular^®^ cornea epithelial model that was successfully validated^9^. In our approach, a similar anatomy as the native cornea was achieved by reducing the culture time of RHE to eleven days. During this time, we could show that the model has not formed cornified layers and has a significantly reduced barrier. Nevertheless, the anatomical differences between skin and cornea are caused by a significantly different genetic expression profile^17^. Moreover, involucrin as a substrate for transglutaminases is expressed in the upper layers and loricrin in the stratum corneum^18^. Although the cornea has a comparable pattern for keratin 14, differentiated cornea epithelium comprises a different network of keratin 3 and 12 instead of keratin 1 in the epidermis^19^. Furthermore, loricrin is found throughout the cornea epithelium and, thus, indicating a different function in cornea as described in epidermis^20^. The basic anatomy between the mRHE and the RCE was comparable in our models, however the marker pattern of skin derived cells still mimicked the one of skin. Equally cornea derived cells showed the pattern found in native human cornea and cornea-specific markers such as keratin 3 and 12 were visible. Also, the distribution of the cornified envelope protein loricrin demonstrated that the cornification is different to the mRHE and RHE (Fig. 1).

To test the hypothesis that the anatomic differences translate to a different tissue barrier, an ET_50_ assay was performed. As expected, the cornified RHE can withstand a hazardous substance much longer than the non-cornified mRHE and RCE, since the Triton X-100 can directly interact with the living cells and does not have to pass the physical barrier of the stratum corneum. For the RCE and the mRHE, no significant difference of the ET_50_ value in the amount of metabolically active cells can be observed, implying a similar barrier of both models. However, employing impedance spectroscopy, a clear difference can be seen. Impedance spectroscopy measures the complex alternating current resistance over a defined frequency spectrum and was employed as a highly sensitive method to assess the influence of different substances on the barrier function of tissue models^12^. An integral part of the previous published method was the mathematical model to derive quantitative data from the impedance spectra. Therefore, a simulation was run based on an equivalent circuit to gain deeper insight into the process involved given the biological reaction. The analysis revealed that the thickness of the model correlates with the capacitance. However, no benefit could be detected in the identification of hazardous substances compared to the here defined TEER_1000 Hz_ (Supplementary Figs 2–4). Although the model allows the precise determination of electrical parameters such as the capacitance or the ohmic resistance, skilled personal and specialized programs are needed to run the simulation. Since a toxicological test should be based on readably accessible technologies that allow a board dissemination of the test method, we developed a novel readout parameter based on the impedance at one defined frequency. The best predictive capability and robustness could be achieved at 1000 Hz, we used the TEER value at this frequency as the readout parameter. The TEER_1000Hz_ was chosen deliberately to ensure an easy use of the test method around the globe, without the need of a complex mathematical fit. Standard measuring systems such as the EVOM system that measures at 12.5 Hz were developed to measure the electrical properties of a single cell layer. Our data shows that at this frequency the predictive capability for three-dimensional tissues is limited as the difference between the unseeded membrane and a mature model is only 19.9 dB (9.9 Ω), whereas for the TEER at 1000 Hz a difference of 36.6 dB (67.7 Ω) can be achieved. Using this method, the RCE model reached a TEER_1000 Hz_ value of only 231 Ωcm^2^ that is significantly lower than the values for the mRHE with 4422 Ωcm^2^ and the RHE with 9268 Ωcm^2^.

Thus, our data suggests that there is a considerable difference between barrier function and marker expression pattern between an *in vitro* model created from skin or cornea cells. However, to the best of our knowledge there is a lack of data if these differences translate to different predictive capacities in an eye irritation assay. Thus, we used all models and compared the performance in an eye irritation assay based on OECD test guideline 492. The test substances were selected from all GHS categories to show feasibility of the test method. Toluene and citric acid were added as unharmonized test substances to provide more insight into their classification and to pose a challenge for the newly developed test method. Since the intention of this study was to test the feasibility of the new impedance-based assay, we limited the number of test substances to nine but suggest to validate the method to evaluate the newly 80 reference chemicals published by the consortium for *in vitro* eye irritation testing^21^.

In the MTT-based eye irritation test, the RCE came closest to distinguish between irritant and non-irritants. Significant changes could be observed between no category against category 1 and a tendency for category 2. This makes the RCE a suitable model for a MTT-based test procedure comparable to the OECD TG 492^9^. Yet, due to donor variances there is still potential for improvement in the selection of donor material. The mRHE could identify category 1 against no category, which is insufficient for safety assessment, detecting non-irritating chemicals. Nevertheless, the OECD TG 492 proves that an epidermal keratinocyte-derived model can be employed. This difference between our model and the published procedure may be because of the smaller selection of test substances in this work. However, the focus of the study was on non-harmonized substances such as citric acid and toluene to get new insights in their categorization but complicating the interpretation^22^. The RHE was not able to distinguish between any categories in a MTT analysis. This supports the hypothesis that the cell origin, while leading to a different marker expression in the model, does not play a deciding role in the classification of substances for eye irritation. This might be different in regard to drug transport studies or metabolism of drugs.

The current *in vitro* eye irritation assays basically follow the experimental approach developed for the identification of skin irritants and skin corrosives. After the application of a test substance the model is measured via a MTT assay and categorized depending on viability normalized to the negative control. However, the classification of eye irritants also require determination of how persistent a reaction is or, in other words, if a caused effect is reversible or not. In the Draize eye test the discrimination between category 2 reversible and category 1 irreversible effects is done by monitoring the rabbit’s eye over a period of 21 days. In case the damage is reversible on day 7 or day 21, the test substance is categorized as 2B or 2A. To test for reversible effects, standard destructive colorimetric assays are not best suited since they lack the possibility to measure the same tissue repeatedly over different time periods and do only take into account effects within the metabolically highly active basal cell layers. As a complementary method impedance spectroscopy could refine test results with the possibility to non-destructively assess also mild effects in the upper cell layers. For instance, it can detect the loss of cell-cell interactions, thereby identifying more sensitive changes caused by the tested chemical. To test this hypothesis, we determined the TEER_1000 Hz_ value before substance application, and right before MTT-measurements and normalized these values to the negative control.

The TEER measurements demonstrated that the electrical analysis allows the detection of smaller differences between the models in comparison to the MTT-based ET_50_ barrier assay. First, the TEER_1000 Hz_ of category 2 substances ethanol and acetone dropped below 45% in the mRHE and RHE, showing an irritative effect in comparison to the MTT-based evaluation. Second, the category 1 substances dropped below 5% and thus even lower than the category 2 substances. Therefore, the ability to distinguish between the categories with the mRHE and the RHE models was improved. The differences for the RCE between the categories were smaller and more heterogeneous. The barrier value was small and the differences were possibly surpassed by donor variances. Nonetheless, this demonstrates the additional value of employing not only a MTT assay but implementing also a more sensitive impedance measurement to improve the test procedure (Fig. 4).

To evaluate all GHS categories for eye irritation, it is important to understand the drivers of eye irritation in each category. Category 2 has to be evaluated by monitoring the rabbit’s eye over 21 days, to determine the reversibility of an effect. In addition, the majority of category 1 chemicals (65%) were classified solely on persistence effects, meaning that the quality of the injury was second to the persistence^3^. This highlights the importance of an analysis which is able to measure non-destructively repeated measurements. Because the TEER measurement allows for repeated measurements of the same model, similar to the observation of the eye in the Draize eye test, it is possible to evaluate reversible effects over time (Fig. 5). Hence, we developed a test procedure, in which we measured the TEER_1000 Hz_ not only before and after test substance application but also subsequently over a post incubation time of 11 day. Since the mRHE model showed the most promising results in the previous test, we employed only this model for the impedance based recovery assay. For each category clear progression patterns of reversible or irreversible effects can be identified by the repeated TEER measurements. Substances that do not need categorization stay around 100% of the control over 11 days, showing no effect. A downward trend after the application, still above 60%, was observable probably caused by the test procedures including multiple washing steps that were already found to influence the barrier. The TEER_1000 Hz_ of category 1 substances decreased the barrier to less than 6% after the substance application. Subsequently, the challenged tissues did not show any sign of recovery and the barrier stayed below 6% and did not increase anymore. Category 2 substances also show a clear decrease after application below 60%, identifying an irritative effect, but in contrast to category 1 substances the TEER_1000 Hz_ increases again over time above 50% showing a regeneration of the barrier and the reversibility of the effect.

**Figure 6:**
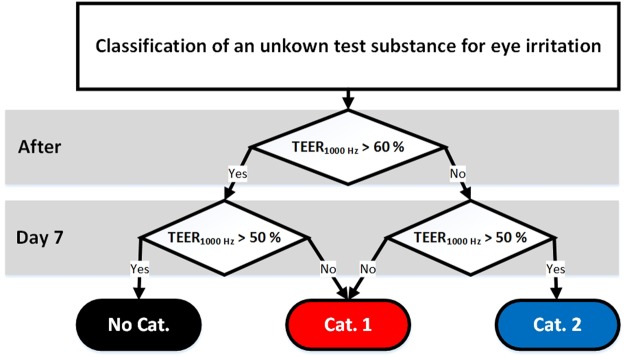
TEER_1000 Hz_ prediction model for the classification of substances according to the GHS categories for eye irritation.

Using this data, we developed a prediction model that uses two time points for the categorization into the three GHS categories (Fig. 6). Directly after the application a 60% cut-off value is used to discriminate not categorized substances versus category 2 and category 1 substances. On day 7 an impedance of 50% is used to distinguish category 2 and category 1 substances. Substances that allow a recovery over 50% are classified as category 2 substances. Effects that persist over more than 7 days indicate strong eye damage and thus the categorization in category 1. In case a substance leads to an impedance value over 60% directly after the application but to a value below 60% on day 7, a persistent effect over time can be assumed that leads to the categorization in category 1.

Employing this prediction model the performed test scores 78% accuracy, making it the first *in vitro* test to distinguish all GHS categories for eye irritation in one test (Table 2). Moreover, we could achieve a reproducibility between the test runs of 88.9% with glycerol being the only exception where we classified the substance as category 2 in one run and no-category in two runs. Although toluene and citrate are partly classified as no category substances, this test suggests a much stronger irritative capacity of both substances. This finding is also supported by several studies that show strong irritative effects of toluene and citrate^23–25^. In addition, toluene is categorized as a skin irritant by the European Chemcials Agency. Taking this into consideration, the test method presented here yields an accuracy of 100%.

**Table 1:** List of test substances used in the eye irritation test.

Test substance	CAS-Number	UN GHS category
Glycerol	56-81-5	No category
Potassium tetrafluorobate	14075-53-7	No category
Toluene	108-88-3	No category
Acetone	67-64-1	Category 2
Ethanol	64-17-5	Category 2
Citrate	77-92-9	Category 2
Imidazole	288-32-4	Category 1
10% Benzalkonium chloride	8001-54-5	Category 1
15% Sodium dodecyl sulfate	151-21-3	Category 1

For all substances the respective number of the Chemical Abstracts Service (CAS-number) and the classification according to UN GHS are provided. For citrate and toluene, the category was chosen based on the majority of reports submitted to the European Chemicals Agency since no harmonized classification is available.

**Table 2:** Categorization of tested substances according to the TEER_1000 Hz_ prediction model.

Test substance	After	Day 7	Prediction	GHS
Glycerol	>60%	>50%	No cat.	No cat.
Potassium tetrafluorobate	>60%	>50%	No cat.	No cat.
Toluene	>60%	<50%	Cat. 1	No cat.
Acetone	<60%	>50%	Cat. 2	Cat. 2
Ethanol	<60%	>50%	Cat. 2	Cat. 2
Citrate	<60%	<50%	Cat. 1	Cat. 2
Imidazole	<60%	<50%	Cat. 1	Cat. 1
Benzalkonium chloride	<60%	<50%	Cat. 1	Cat. 1
Sodium dodecyl sulfate	<60%	<50%	Cat. 1	Cat. 1

Finally, this methods holds the potential to subcategorize substances as category 2A and 2B by identifying more or a faster regeneration between substances of category 2A and 2B. However, if this is necessary is open for discussion since the regulatory bodies have different opinions on the matter. The European Union for example did not include the subcategories. TEER measurements at 12.5 Hz have shown to be a profound method to evaluate 118 chemicals for eye irritation^23^. In this study, however only three minutes after the application were measured not allowing for further evaluation of the reversibility of effects. In addition, we could show that the TEER measurements at 1000 Hz are more sensitive to evaluate corneal epithelial models. To our knowledge only the porcine corneal ocular reversibility assay can distinguish between the GHS categories for eye irritation in one test^26^. However, the test method uses porcine eyes, which could lead again to species differences and still relies on animal material. In addition, the procine corneal ocular reversibility assay is not a validated method and has not been adopted into the OECD guidelines.

The systematic analysis of *in vitro* models derived from corneal and epidermal epithelial cells identifies anatomical and molecular differences. Nonetheless, the RCE and the mRHE translate into appropriate models for the identification of hazardous chemicals for eye irritation. Furthermore, the implementation of impedance spectroscopy led to a more precise analysis tool compared to a MTT-based test. Finally, by employing this non-destructive measurement tool repeated measurements allow for the evaluation of recovery or persistence of effects. This enables for the first time the identification of all three GHS categories for eye irritation in one single test. The next step could include a larger amount of test substances to identify possible limitations of the TEER measurements and conclude in the validation of an improved OECD TG 492 to make the animal-based Draize eye test obsolete.

## Methods

### Human material

All experiments were conducted in compliance with the rules for investigation on human subjects, as defined in the Declaration of Helsinki. Informed consent was obtained from all subjects prior to the study. Human corneal cells were isolated from spare limbal rings and from corneas that failed the quality criteria for clinical use in accordance and with the approval of the local ethics committee (Ethik-Kommission der Universität Würzburg, approval number 182/10) and the informed consent of the patients for the study participation. Corneal biopsies were provided by the eye clinic of the Universitätsklinikum Würzburg (Würzburg, Germany). Human epidermal keratinocytes were isolated from foreskin biopsies of 2–5 year old donors in accordance and with the approval of the local ethics committee (Ethik-Kommission der Universität Würzburg, approval number 182/10) and the informed consent of the patients or their guardians for the study participation. Foreskin biopsies were provided by the Klinikum Frankfurt Höchst (Frankfurt, Germany). No tissues were procured from prisoners.

### Cell isolation and culture

Human epidermal keratinocytes were isolated from foreskin using a well-established protocol described previously^27^. In brief, biopsies were washed, minced and digested with dispase [2 U/ml] (Life technologies, Germany) for 18 hours at 4 °C to dissociate the epidermis from the dermis. Thereafter, the epidermis was trypsinized (Thermo Fisher Scientific, Waltham, MA USA) to generate single cell suspensions. The epidermal keratinocytes were cultured in EpiLife® medium containing human keratinocyte growth supplement (Both from Thermo Fisher Scientific, Waltham, MA USA)).

Corneal tissues were transferred and washed in a petri dish with phosphate-buffered saline. Subsequently, the cornea was cut into horizontal stripes of about 2–3 mm and put in a petri dish with dispase [2 U/ml] (Thermo Fisher Scientific, Waltham, MA USA)) for 18 hours at 4 °C. The epithelium was stripped off the central cornea to the limbus with forceps and collected in a new petri dish with fresh phosphate-buffered saline. The epithelial sheets were centrifuged and reduced to small pieces by pipetting for cell seeding. The corneal epithelium was cultured in corneal epithelial cell medium (LGC Standards, Wesel, Germany).

### Tissue models

The RHE was generated as previously described^28^. Briefly, human epidermal keratinocytes were seeded on cell culture inserts with a polycarbonate membrane (0.4 µm pore size, Merck, Darmstadt, Germany) at a cell density of 5 × 10^5^ cells/cm^2^ in 150 μl EpiLife® supplemented with human keratinocyte growth supplement and 1.5 mM CaCl_2_ (Sigma-Aldrich, Munich, Germany). Medium was changed after 24 hours to EpiLife® air-liquid-interface medium, additionally containing 73 μg/ml L-ascorbic acid 2-phosphate and 10 ng/ml keratinocyte growth factor (both Sigma-Aldrich, Munich, Germany). The RHE models were cultured for 20 days. In order to generate the mRHE, the culture period was reduced to 11 days. The RCE was generated from human corneal epithelial cells. The cells were seeded as described above with a cell density of 2.5 × 10^5^ cells/cm^2^ and cultured for 11 days.

### Histology

Native tissue and tissue models were fixed in Roti®-Histofix (Carl Roth, Karlsruhe, Germany) for 2 hours before generating histological cross-sections of 4 µm thickness. As an overview of general histological features, tissue slides were stained with hematoxylin and eosin (H&E; Morphisto, Frankfurt am Main, Germany). For immunohistochemical staining tissue slides were rehydrated and blocked with 5% donkey serum for 30 minutes. Subsequently, the primary antibodies cytokeratin 3/12 (1:100 dilution; Bioss, MA USA), cytokeratin 1 (1:1000 dilution; Abcam, Cambridge, United Kingdom), cytokeratin 14 (1:100 dilution; Sigma-Aldrich, Munich, Germany), involucrin (1:200 dilution; Thermo Scientific Fisher, MA USA) and loricrin (1:500 dilution; Abcam, Cambridge, United Kingdom) were added and incubated at 4 °C overnight. After washing, the slides were exposed to the matching secondary antibodies Alexa Fluor® 555 donkey anti-mouse or Alexa Fluor® 555 donkey anti-rabbit (1:400 dilution; Thermo Fisher Scientific, Waltham, MA USA) for 30 minutes at room temperature. The sections were covered with Fluoromount-G DAPI (Thermo Fisher Scientific, Waltham, MA USA) to visualize the cell nuclei.

### Epithelial barrier assay

The epithelial barrier was determined by the exposure time required to reduce cell viability by 50% (ET_50_) as described previously^29^. Briefly, the tissue models were topically exposed to 25 µl of 1% (w/v) Triton X-100 (Sigma-Aldrich, Munich, Germany) at 37 °C for 0.5, 1.5, 3.0, 4.5 and 6 hours to determine the ET_50_ value indicative for the respective barrier. To equally cover the model, the Triton X-100 was frothed up by pipetting. Untreated models served as a negative control. After incubation the models were washed in a beaker with 150 ml phosphate-buffered saline. The models were put into a 24-well plate with 300 µl of 1 mg/ml MTT-solution and incubated for 3 hours at standard culture conditions. To solubilize the blue formazan, the inserts were submersed in 2 ml isopropanol at 4 °C overnight. Finally, the insert membranes were punched and 200 µl of each sample was measured in duplicates in a 96-well plate with a spectrometer (Infinite M200 Pro, Tecan, Männedorf, Switzerland) at a wavelength of 570 nm using isopropanol as blank.

### Impedance spectroscopy measurement

The impedance spectroscopy measurement was performed as previously described^30^. In brief, a sinusoidal electrical current I(f) [A] was generated and the potential difference U(f) [V] was measured by an impedance spectrometer LCR HiTESTER 3522-50 (HIOKI E.E. Corporation, Nagano, Japan) to record the impedance spectra Z(f) of biological samples. A custom-made user interface, programmed in LabVIEW (National Instruments, Austin, TX USA) calculated Z(f) according to the equation Z(f) = U(f)/I(f).

RHE, mRHE, and RCE models were measured in a 24-well plate (Brand, Wertheim, Germany) with an in-house-developed measuring chamber (Supplementary Fig. 1). The insert and the well were filled with 0.5 ml EpiLife® basal medium (Thermo Fisher Scientific, Waltham, MA USA) to ensure contact with the electrodes. For each well, impedance was measured over a frequency range from 1 Hz to 100 kHz at 40 logarithmically distributed sampling points.

### Eye irritation test

The eye irritation test was based on the test protocol of the OECD test guideline 492 to ensure the high quality standards for the validation of a new test system^9^. In short, three independent test runs with two technical duplicates were performed for each model. The tissue models were measured via impedance spectroscopy before the eye irritation test. Models were then moisturized with 20 µl phosphate-buffered saline. 50 µl of liquid test substances was applied in duplicates for 30 minutes, washed three times, submersed in a 12-well plate with 2.5 ml culture medium for 12 minutes and let to rest under normal culture conditions for 120 minutes. 50 mg of solid test substances was applied in duplicates for 6 hours, washed three times, submersed in a 12-well plate with 2.5 ml culture medium for 25 minutes and let to rest under normal culture conditions for 18 hours. Phosphate-buffered saline was employed as negative control and 5% sodium dodecyl sulfate as positive control. The impedance of the models was measured again. Subsequently, a MTT-test was performed as described above and measured in duplicates with a spectrometer (Infinite M200 Pro, Tecan, Männedorf, Switzerland) at a wavelength of 570 nm using isopropanol as blank.

### Statistical analysis

Datasets were tested for normality with the D’Agostino-Pearson omnibus test. Based on normality, statistical differences were analyzed by one-way ANOVA or Kruskal-Wallis. Values of p < 0.05 were considered to be significant. Datasets were analyzed in GraphPad PRISM 6 (GraphPad Software Inc., La Jolla, CA USA).

## Electronic supplementary material

Supplementary Information

## Data Availability

The datasets generated during and/or analysed during the current study are available from the corresponding author on reasonable request.
