# Supplementary material for: Replacing the Draize eye test: Impedance spectroscopy as a 3R method to discriminate between all GHS categories for eye irritation
Source: Sci Rep. 2018 Oct 9;8:15049. doi: 10.1038/s41598-018-33118-2 (PMC6177390; doi:10.1038/s41598-018-33118-2)
Supplement: Supplementary file 1 — Supplementary Information [file 41598_2018_33118_MOESM1_ESM.pdf]

Replacing the Draize eye test: Impedance spectroscopy as a 3R method to discriminate between all GHS categories for eye irritation

C. Lotz[1*], L. Engelhardt[1], F. F. Schmid[2], J. Hansmann[1,2], H. Walles[1,2], F. Groeber-Becker[2]

1 Department Tissue Engineering & Regenerative Medicine (TERM), University Hospital Würzburg; Würzburg 97070, Germany

2 Translational Center for Regenerative Therapies, Fraunhofer Institute for Silicate Research; Würzburg 97070, Germany

Supplementary Information

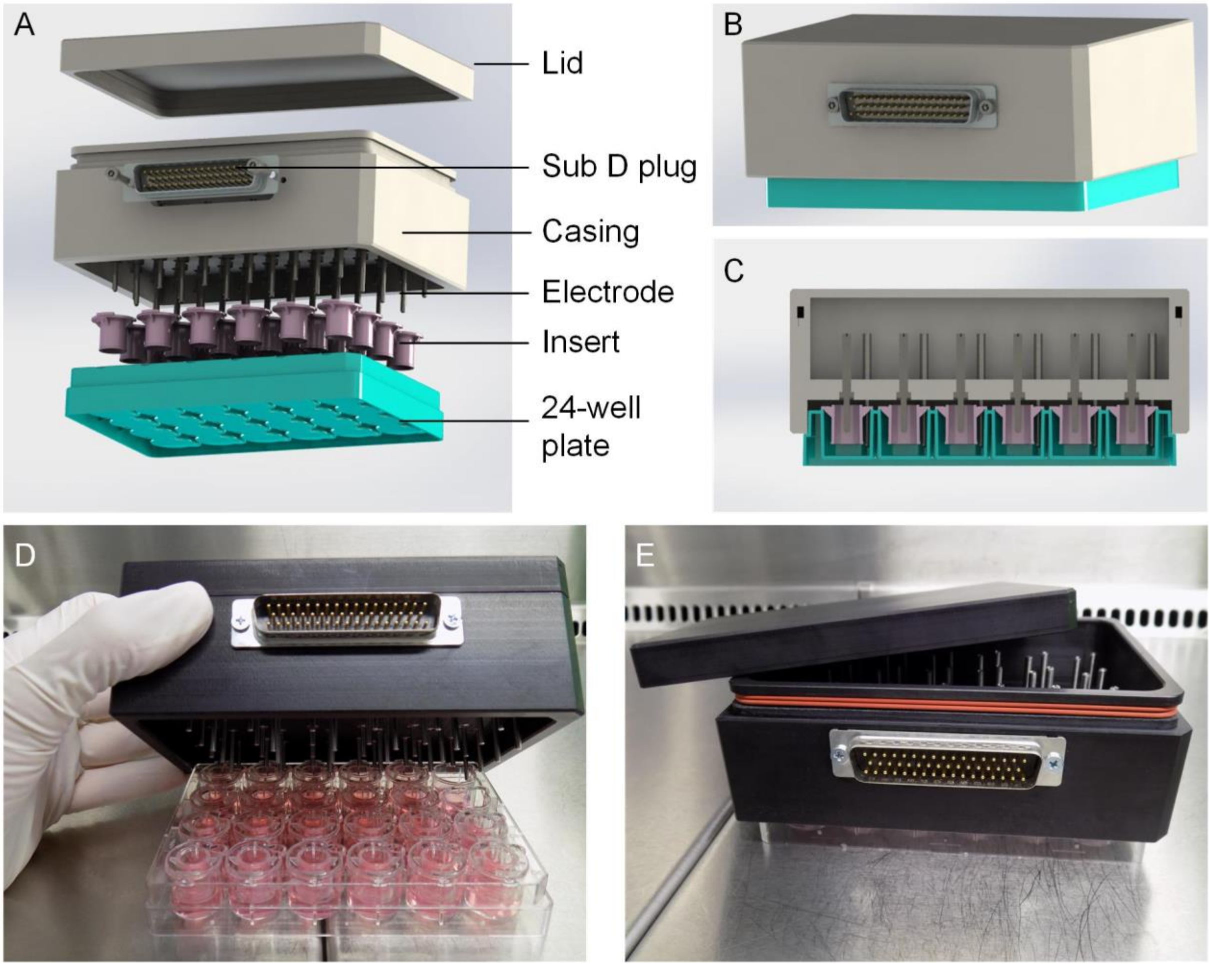

Supplementary Fig 1: Impedance measurement chamber. (A) Computer-aided design model of the imped-ance measurement chamber with inserts and 24-well plate in an exploded view. (B) Computer-aided design model of the joint configuration (C). Computer-aided design model of the joint configuration in longitudinal section. (D) Constructed impedance measuring chamber front view and (E) from above.

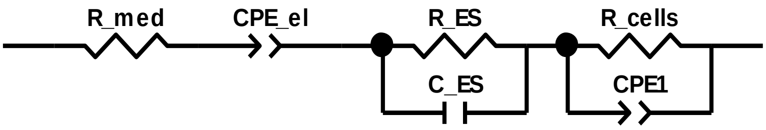

Supplementary Fig 2: Equivalent circuit diagram for the fit of the impedance data. Resistance of the medium (R_med), constant phase element of the electrode (CPE_el), resistance of the electrode (R_ES), capacitance of the electrode (C_ES), Resistance of the model (R_cells) and constant phase element of the model (CPE1).

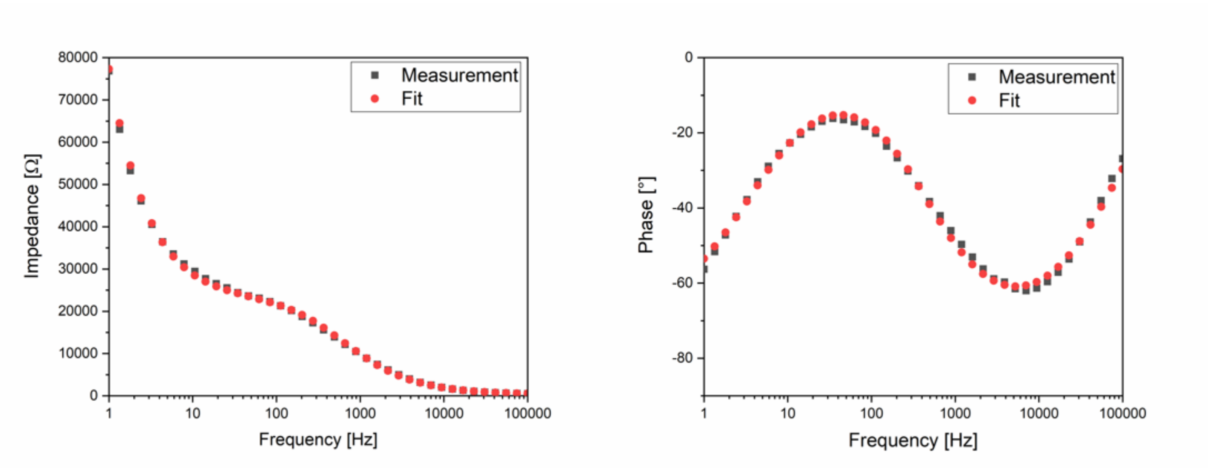

Supplementary Fig 3: Simulation of the impedance data were performed using the software NOVA 2.1 (Metrohm Autolab, Utrecht, The Netherlands). Parameters for the electrical equivalent circuit were determined by the program via a nonlinear least-squares optimization method. Exemplary fit of a mRHE.

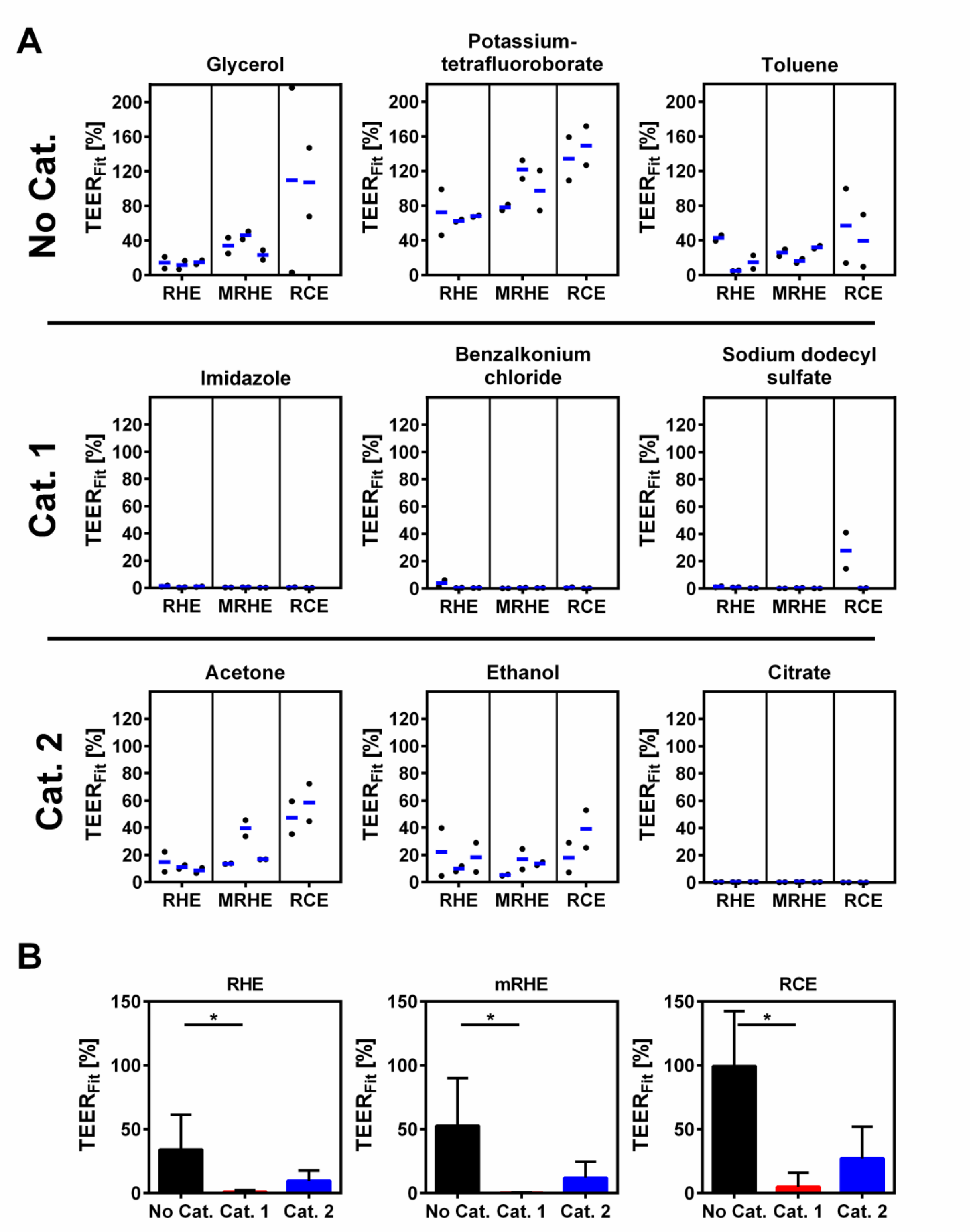

Supplementary Fig 4: The fitted TEER ($TEER_{Fit}$) in regard to discriminate between the GHS categories of eye irritation. The $TEER_{Fit}$ does not improve the ability to distinguish between the GHS categories.
